# Locus of control as a personal coping resource of a sportsman

**DOI:** 10.1192/j.eurpsy.2023.2159

**Published:** 2023-07-19

**Authors:** S. Fedorchuk, T. Petrovska, I. Kohut, O. Hanaha, L. Arnautova

**Affiliations:** National University of Ukraine on Physical Education and Sport, Kyiv, Ukraine

## Abstract

**Introduction:**

In sports psychology, the issue of finding resources to overcome stress remains relevant at present. Currently, the priority is the search for personal resources that can help overcome difficult life situations. Currently, the priority is the search for personal resources that can help overcome difficult life situations. Research by many psychologists (Folkman S., Hobfoll S., Haan N.A., Heim E., Lazarus R., Moos R.N., Schaefer C., Grin O.R., Dementiy L.I., Kalnysh V.V., Tukaiev S.V., Khazova S.A. et al) is devoted to this topic. Among the coping resources, the authors single out motivation, locus of control, resilience, self-control, purposefulness, outlook, intelligence, etc.

**Objectives:**

The purpose of the study is the analysis of literary sources regarding the study of the locus of control as a personal coping resource of an athlete.

**Methods:**

To realize the goal of the work, the following were used: theoretical analysis and generalization of literary sources and Internet data.

**Results:**

According to the results of research by domestic scientists, the locus of control and responsibility can act as a coping resource. It was found that the internal locus of control is associated with a low level of anxiety, and the external one with a high one; in addition, an inverse relationship between the locus of control and the level of neuroticism was found (Dementiy, 2005). This indicates that respondents with an internal locus of control are more stable in their behavior in situations that provoke anxiety and actualize personal anxiety. Internality allows a person to maintain a sense of control over the situation and his condition. It has been proven that the relationship between the locus of control and the state of anxiety depends on volitional self-control. However, along with this, the phenomenon of “heaviness of responsibility” is known in the scientific literature, which manifests itself in a sharp increase in anxiety with an internal locus of control. This may indicate that internality “loses” its resourcefulness under certain conditions: relationships between internality and anxiety are evident only at a low level of self-control. In the case when a person has developed self-control, such a connection is broken.

**Conclusions:**

The generally accepted structure of personal resources, which determine the effective behavior of an individual under conditions of stress, has not yet been formed. Locus of control can act as a coping resource in the structure of self-regulation of an individual.

**Disclosure of Interest:**

None Declared

